# Research on differences in scalp aging characteristics and visual attention between genders in the Chinese population

**DOI:** 10.3389/fmed.2026.1744737

**Published:** 2026-02-02

**Authors:** Susu Ding, Dangdang Cheng, Rong Qi, Feifei Wang

**Affiliations:** 1 Shanghai Jiyan Biomedical Development Co., Ltd., Shanghai, China; 2 Yunnan Botanee Bio-technology Group Co., Ltd., Yunnan, China; 3 Yunnan Characteristic Plant Extraction Laboratory Co., Ltd., Yunnan, China

**Keywords:** eye-tracking, gender difference, non-invasiveinstrumental measurement, scalp aging, subjective questionnaires, visual attention

## Abstract

**Objective:**

This study aimed to explore the differences in characteristics of scalp aging and visual attention across genders in a Chinese population.

**Methods:**

This study recruited 79 Han Chinese participants aged 31–47 years from Shanghai, China. Using a combination of non-invasive instrumental measurements, eye-tracking technology, and subjective questionnaires, we analyzed scalp aging manifestations through three physiological dimensions—barrier function, microecology, and scalp skin color—while examining visual attention patterns toward scalp aging features through eye-tracking and assessing subjective cognitive and emotional responses via questionnaires.

**Results:**

The results revealed no significant gender differences in scalp barrier function. Instrumental measurements showed no notable differences in stratum corneum moisture content or transepidermal water loss (TEWL) between genders, and subjective evaluations of moisture, glossiness, greasiness, and tightness also showed no significant variations. However, significant gender differences were observed in scalp microecology: women exhibited higher dandruff area proportion and pH, along with more severe subjective concerns regarding hair loss. In terms of scalp skin color, men had higher a^*^ values, though no significant gender difference was reported in subjective perceptions of scalp redness. Regarding visual attention, eye-tracking data indicated distinct gender-based patterns: women focused more persistently on dandruff and hair loss, allocating greater cognitive resources to these features, whereas men exhibited more concentrated and frequent attention to gray hair and oily scalp. Subjectively, the majority of participants believed that scalp aging negatively impacts personal attractiveness and reported high levels of concern.

**Conclusion:**

This study revealed significant gender-based differences in physiological characteristics and visual attention patterns associated with scalp aging in the Han Chinese population in Shanghai, China. These findings provide a scientific basis for understanding scalp aging and for developing related products.

## 1 Introduction

With the accelerating aging population, skin aging has increasingly become a focal issue of public concern (1–3). As a visible indicator of systemic aging, the skin not only serves essential barrier and defensive functions but also carries sociopsychological significance as a symbol of “external attractiveness” (4). Skin aging is a complex biological process that is categorized into intrinsic aging, driven by genetic factors, hormonal changes, and mitochondrial dysfunction, and extrinsic aging, primarily caused by external factors such as UV radiation, air pollution, tobacco smoke, and lifestyle (5–7). The “primary site” of aging is not limited to the face: the scalp, which covers approximately two-thirds of the head and facial skin, also undergoes a similar aging process, influenced by genetic regulation, oxidative stress, inflammatory responses, microcirculatory impairment, and follicular degeneration (8–10). Furthermore, with the growing emphasis on self-care and personal wellbeing (“self-pleasure ideology”), public attention toward scalp appearance is increasing. Nevertheless, visual attention research focusing on the external manifestations of scalp aging remains limited.

In recent years, eye-tracking technology has emerged as a non-invasive tool that captures ocular behaviors—such as gaze trajectories, fixation duration, and pupil variations—to infer underlying cognitive processes and psychological states, such as emotional responses (11–15). Owing to these advantages, it has been widely applied across domains such as interface optimization in human–computer interaction (16), attention monitoring in intelligent driving (17), and auxiliary diagnostics in healthcare (18). Despite these advancements, its application in the cosmetics industry remains limited, particularly in evaluating the visual characteristics of scalp aging. Similarly, in the field of cosmetics, eye-tracking can quantify consumers’ visual attention to packaging, advertising, and usage (19–21), which in turn can assist formulation optimization and precision marketing. However, research on visual attention to aging features of the facial and scalp skin (e.g., discoloration, wrinkles, dandruff, and hair loss) remains limited. The present study used eye-tracking to examine how participants visually attend to scalp aging features, thereby providing an objective basis for understanding these characteristics.

According to the Expert Consensus on Scalp Anti-Aging in China, objective manifestations of scalp aging include impaired barrier function (e.g., dryness, flaking, and excessive sebum), thinning and laxity of the scalp, pruritus, inflammation, cutaneous neoplasms, graying and thinning of hair, and hair loss (22). While previous studies have largely focused on age-related changes in women (23, 24), gender differences remain underexplored. Evidence from Maymone suggests that signs of scalp aging typically become clear after the age of 30 (9, 25). Therefore, this study targets the 31–47 age group and employs a multimodal approach combining instrumental measurements and eye-tracking to comprehensively assess gender-based differences in scalp aging characteristics and visual attention patterns among Chinese adults. The findings aim to provide new insights and scientific support for efficacy evaluation in cosmetic development, thereby enriching research in this emerging field.

## 2 Materials and methods

### 2.1 Participants

This study recruited a total of 79 healthy male and female participants aged 31–47 years in Shanghai, China, in May 2025. The inclusion criteria were as follows: Han Chinese ethnicity with continuous residency in Shanghai for ≥2 years; an intact scalp without conditions such as breakouts, acne, scars, birthmarks, pigmented nevi, or inflammation; no chemical hair treatments (e.g., dyeing, perming, or styling) within the past 3 months; and normal or corrected-to-normal vision, with no color blindness or color weakness. The exclusion criteria included pregnancy, lactation, or plans for pregnancy in the near future; severe androgenic alopecia, alopecia areata, inflammatory scarring alopecia, or other scalp or hair disorders; diagnosed psychiatric or psychological conditions; chronic sleep disorders or emotional dysregulation; high physical sensitivity; or a history of hair transplant procedures. The study strictly adhered to the principles of the Declaration of Helsinki, and all participants provided written informed consent after being fully informed of the study details. The protocol was approved by the Jiyan Ethics Research Committee (No.: JYE20250503).

### 2.2 Instruments

A range of non-invasive instruments was employed to assess scalp biophysical properties: Skin-pH-Meter (CK, Germany), Sebumeter SM 815 (CK, Germany), Skin-Glossymeter (CK, Germany), Indentometer IDM 800 (CK, Germany), Visioscan® VC 20plus (CK, Germany), D-Squame Pressure Instrument D500 (CuDerm, USA), Vapometer Nano (Delfin, Finland), PhotoMax Pro (DMS, Austria), Image-Pro Plus (IPP; Media Cybernetics, USA), DermaLab® Combo (Cortex, Denmark), and Tobii Pro Fusion (Tobii, Sweden).

### 2.3 Study design

Non-invasive instruments were first used to collect physiological parameters of the participants’ scalps, followed by the completion of self-assessment questionnaires. Finally, an eye-tracking task and a visual perception questionnaire were administered.

#### 2.3.1 Collection of scalp aging characteristics

Before the participants’ visit, they were required not to wash their hair for 48 ± 4 h. After arriving at the laboratory, the ambient temperature was 20–22 °C, and the humidity ranged from 40 to 60%. After balancing for 30 min, a total of 10 physiological parameters were collected. The specific parameters and measurement conditions are shown in Table 1.

**Table 1:** Measurement of scalp physiological parameters.

Instrument	Parameters	Definitions	Measurement area	Collection times
Ultrasound DermaLab®Combo	Hydration	The larger the measured value, the higher the moisture content of the stratum corneum of the skin	The parietal bone spins	3
	Temperature	The larger the measured value, the higher the skin temperature	The parietal bone spins	3
Vapometer	TEWL	The smaller the measured value, the less water evaporates from the skin within a unit of time	The parietal bone spins	2
Indentometer IDM800	The softness of the skin	The larger the measured value is, the softer the skin is	The parietal bone spins	3
Skin-pH-Meter pH 905	pH	The smaller the measured value, the more acidic the skin is	The parietal bone spins	3
Skin-glossymeter	Glossiness	The larger the measured value, the more lustrous the skin is	The parietal bone spins	3
Sebumeter SM 815	Sebum	The larger the measured value, the higher the sebum	The parietal bone spins	2
Visioscan® VC 20plus	Stratum corneum peeling index	The larger the analyzed value, the higher the proportion of dandruff	Bilateral temporal regions	1^#^
PhotoMax Pro/IPP	*a* ^*^	The larger the analysis value, the redder the skin	The parietal bone spins	2^#^
	*b* ^*^	The larger the analysis value, the yellower the skin		

^#^Indicates image acquisition. The asterisk (*) is the standard symbol for the CIE L*a*b* color space.

##### 2.3.1.1 Self-assessment

The participants conducted self-evaluations in front of a mirror under lighting with the same color temperature and completed the questionnaires simultaneously. The questionnaire primarily included items on scalp moisture, dandruff, scalp oil, scalp tightness, scalp itching, scalp softness, scalp elasticity, scalp color, scalp glossiness, hair loss, and overall hair density. The scoring system adopted a scale ranging from 1 to 5 (1 point = very poor, 2 points = poor, 3 points = average, 4 points = good, and 5 points = very good). For each item, the scoring criteria were described in detail. For example, for hair loss, 1 point indicated very poor (excessive and very obvious hair loss), whereas 5 points indicated very good (healthy hair with no hair loss).

#### 2.3.2 Eye-tracking task

Participants were seated in a quiet environment with controlled, moderate lighting. The experiment was conducted using a 24-in laptop (16,9 aspect ratio, 1920 × 1,080 resolution) for visual stimulus presentation. Prior to the task, the researcher explained the procedure and precautions to each participant. An eye-tracker calibration was performed to ensure accurate gaze recording. During calibration, participants were instructed to maintain a distance of 60 cm from the screen and focus on a central black crosshair target. The target remained visible for 1–3 s before moving horizontally to positions ±10° from the center, where it stayed for an additional 1–2 s.

**Figure 1:**
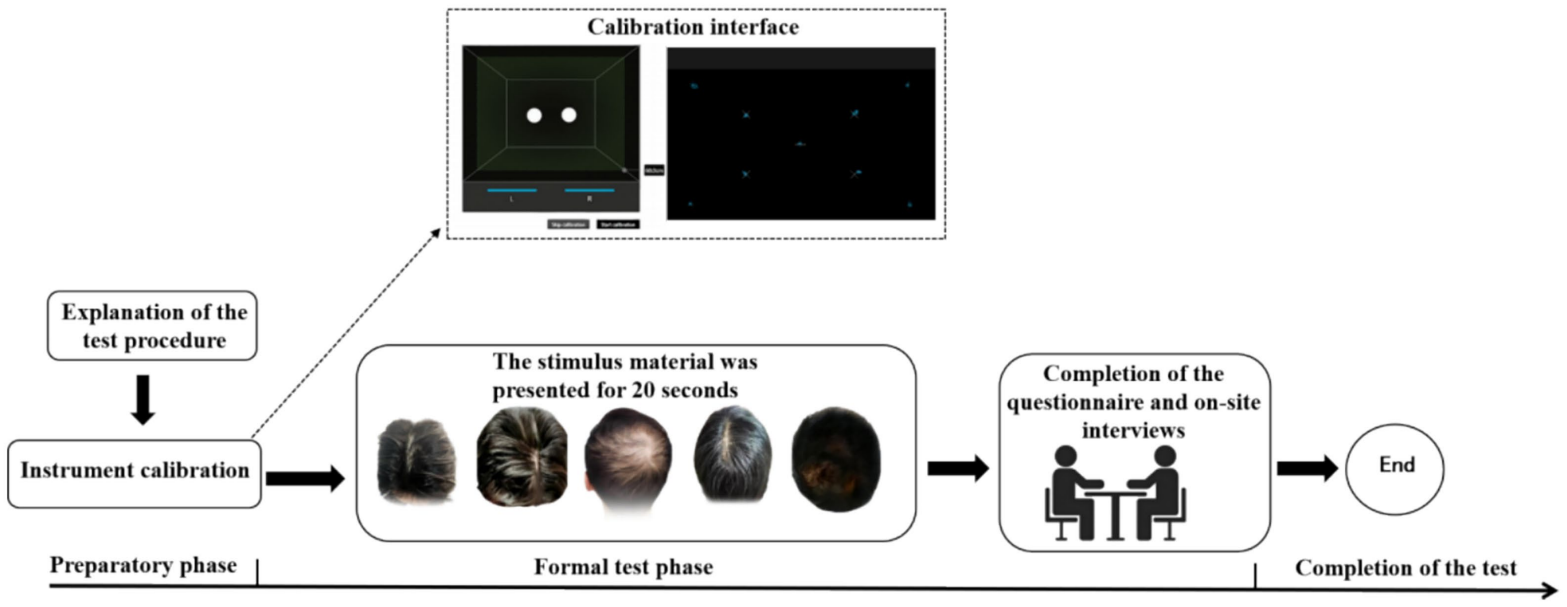
Schematic diagram of eye-tracking.

After successful calibration, a stimulus image displaying five characteristic signs of scalp aging—dandruff, pigmentation, gray hair, oily scalp, and hair loss—was presented on the screen for 20 s. The features were arranged uniformly in a random order to mitigate central bias (14). Subsequently, participants completed a questionnaire and a brief interview aimed at capturing their subjective perceptions of the visual stimuli and their cognitive awareness of aging features, thereby providing insight into their psychological and behavioral responses. The entire session lasted approximately 20 min. A schematic overview of the experimental procedure is provided in Figure 1.

##### 2.3.2.1 Data selection

This study employed key eye-tracking metrics related to visual fixation to examine participants’ visual attention to scalp aging features. The selected indicators included time to first fixation, first fixation duration, total fixation duration within the area of interest (AOI), average fixation duration, visit duration, fixation count within the AOI, and number of visits to the AOI. Definitions and interpretations of each parameter are provided in Table 2. Using the AOI tool in Tobii Pro Lab software, regions of interest were precisely delineated for each scalp feature. The software’s visualization function was then utilized to generate heatmaps, providing intuitive graphical support for analyzing gaze patterns.

**Table 2:** Parameters related to eye-tracking.

Parameters	Description
Time to first fixation in AOI/s	The time required from the start of stimulation in the area of interest to the participant’s first gaze at that area reflects the efficiency of attention captured in that area.
Duration of first fixation in AOI/s	The duration of the first gaze at the area of interest, the longer the time, the more difficult or attractive the information processing is.
Total duration of fixation in AOI/s	The total sum of all gaze times on the area of interest is used to measure the overall visual appeal of a certain area.
Average duration of fixation in AOI/s	The average duration of each gaze within the region of interest reflects the average cognitive processing depth of the participant toward a specific area.
Total duration of visit/s	The total time spent in the area of interest reflects the overall stay time of the participants in a specific area.
Number of fixations in AOI/times	The number of gazes within the same area of interest. The more gazes there are, the higher the degree of attention in the corresponding area.
Number of Visits/times	The number of follow-up visits to the same area of interest reflects the level of visual appeal of that area.

### 2.4 Statistical analysis

SPSS Statistics 26.0 statistical software was used for data analysis. All data are expressed as mean±standard deviation. For instrumental skin measurements between male and female participants, an independent samples *t*-test and a one-way ANCOVA (with age as the covariate) were used. The questionnaire data were analyzed using an independent samples *t*-test and chi-squared test. Ordinal data are presented as the number of cases (percentage). Prior to any independent-samples *t*-test, Shapiro–Wilk normality and Levene’s homogeneity-of-variance tests were performed. If both groups showed a normal distribution and homogeneity of variance, the independent-samples *t*-test was used; if at least one group deviated from normality or variances were unequal, the Mann–Whitney U test (rank-sum test) was applied. The significance level was set at *α* = 0.05.

## 3 Results

### 3.1 Participant characteristics

A total of 79 subjects were included in this study, among whom 31 were men and 48 were women (aged 31–47 years), as shown in Table 3.

**Table 3:** Participant characteristics.

Groups	Number	Average age
Men	31	40.1 ± 3.9
Women	48	41.4 ± 4.0

### 3.2 Differences in physiological parameters related to scalp aging

**Figure 2:**
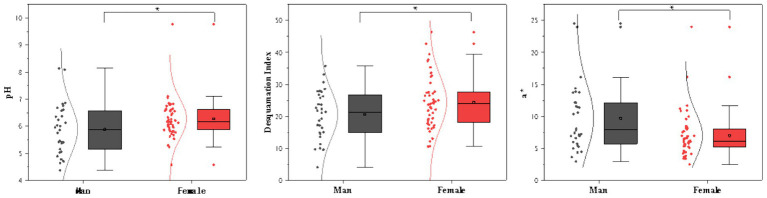
Physiological difference indicators of the scalps between men and women.

Differences in physiological parameters related to scalp aging between the two groups were compared and analyzed. The specific data are presented in Table 4. Based on the results of the difference analysis, a difference graph was generated, as shown in Figure 2.

**Table 4:** Differences in physiological parameters related to scalp aging.

Dimensions	Parameters	$\bar{X}$ ± SD		*P*	*P* (ANCOVA)
		Men	Women		
Skin barrier	Hydration /AU	57.59 ± 32.95	51.55 ± 27.50	0.450	0.250
	TEWL/g/m^2^h	24.08 ± 6.02	23.51 ± 5.87	0.457	0.565
Skin microecology	Temperature/°C	32.01 ± 0.76	32.15 ± 0.60	0.691	0.302
	pH/−	5.88 ± 0.92	6.28 ± 0.73	0.026^*^	0.039^#^
	Sebum /ug/cm^2^	59.32 ± 40.08	43.80 ± 29.15	0.088	0.085
	Proportion of dandruff area/%	20.60 ± 7.49	24.40 ± 8.22	0.042^*^	0.033^#^
	The softness of the skin/mm	1.41 ± 0.69	1.36 ± 0.61	0.972	0.816
	Glossiness/GU	3.57 ± 1.67	3.84 ± 2.83	0.833	0.785
Scalp skin color	a^*^/−	9.71 ± 5.29	6.99 ± 3.60	0.011^*^	0.006^#^
	b^*^/−	10.08 ± 4.83	8.08 ± 2.61	0.151	0.109

^*^Indicates a significant difference, *p* < 0.05. “−” indicates that the data has no specific unit.

^#^Indicates a significant difference, *p* < 0.05 (ANCOVA).

#### 3.2.1 Relationship between scalp aging and barrier

The scalp barrier function is composed of the stratum corneum and the sebaceous film. The “brick-and-mortar” structure of the stratum corneum consists of corneocytes and intercellular lipids, with ceramides helping to prevent water loss and defend against external aggressors, thereby maintaining scalp health (26). Transepidermal water loss (TEWL) is a key indicator for evaluating skin barrier integrity (27). In this study, TEWL values and stratum corneum moisture content were used as two critical parameters to comprehensively assess the barrier function of the participants’ scalps. The results indicate that, within the 31–47 age group characterized by ongoing aging, no significant differences were observed between men and women in barrier-related metrics, suggesting that gender does not exert a substantial influence on scalp barrier function at this stage of aging.

#### 3.2.2 Relationship between scalp aging and microecology

The scalp microecology comprises the microbial communities (such as bacteria, fungi, and viruses) on the scalp surface and around hair follicles, as well as their interactions with the scalp environment (28, 29). Scalp aging is often accompanied by microecological imbalance, with dandruff serving as a prominent external manifestation (30). Its formation is closely associated with the overgrowth of *Malassezia*, which breaks down triglycerides in sebum into free fatty acids, thereby compromising the scalp barrier and triggering inflammation and desquamation (31). Additionally, an elevated scalp pH is linked to microecological dysregulation, as higher pH levels can disrupt the natural barrier and lead to microbial imbalance (32).

To comprehensively evaluate scalp microecology, this study selected dandruff area percentage and pH level as primary indicators, with temperature, sebum, softness, and glossiness as secondary parameters. The results revealed that women exhibited significantly higher dandruff coverage and pH levels than men, with no significant differences observed in the other parameters between the sexes. Related studies (33, 34) also report that women’s forehead and facial pH levels are significantly higher than those of men. These results suggest that significant gender differences exist in scalp aging and microbiome imbalance.

**Figure 3:**
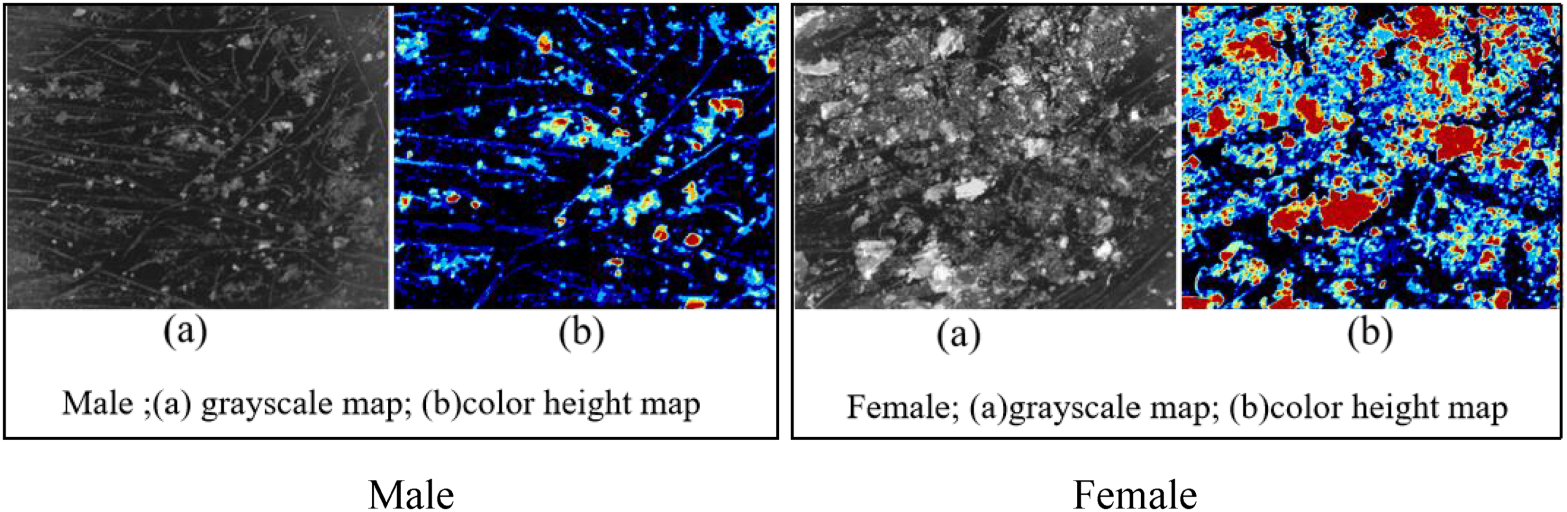
Comparison of dandruff distribution characteristics between men and women.

Figure 3 illustrates the distribution characteristics of dandruff in male and female participants: (a) a grayscale map shows the distribution of dandruff, and (b) a color height map in which red, orange, and light green indicate areas with thicker scaling, while dark blue and light blue represent thinner scaling.

#### 3.2.3 Relationship between scalp aging and scalp skin color

Changes in pigmentation are among the visible manifestations of scalp aging (10, 35). A normal, healthy scalp typically exhibits a bluish-white tone with good elasticity and gloss. Alterations in scalp color—such as excessive sebum secretion, dryness and dullness, abnormal hyperkeratosis, or atrophy—often indicate an aging state (22). In this study, the a^*^ and b^*^ values from the CIE (International Commission on Illumination) standard color system were used as key indicators to evaluate the scalp skin color. The a^*^ value represents the green–red axis, with higher values indicating stronger redness, while the b^*^ value corresponds to the blue–yellow axis, with higher values indicating stronger yellowness (36, 37).

The results showed that, among participants aged 31–47, men had significantly higher a^*^ values than women (*p* < 0.05), whereas no significant gender difference was observed in b^*^ values (*p* > 0.05). This finding indicates that, within this age group, male scalps exhibit a relatively higher degree of redness.

#### 3.2.4 Self-assessment

The self-assessment results of scalp-related indicators for men and women obtained through questionnaire surveys are shown in Table 5.

**Table 5:** Self-assessment results of the subjective questionnaires.

Dimensions	Characteristics	$\bar{X}$ ± SD		*P*
		Men	Women	
Skin barrier	Moisture	2.81 ± 0.54	2.92 ± 1.11	0.145
	Glossiness	2.87 ± 0.92	2.75 ± 0.84	0.566
	Greasiness	2.32 ± 1.01	2.90 ± 1.04	0.565
	Tightness	3.06 ± 0.81	2.79 ± 0.87	0.192
Skin microecology	Burning	3.02 ± 0.33	2.99 ± 0.21	0.533
	Itching	2.68 ± 0.87	2.69 ± 0.95	0.749
	Scurf	3.17 ± 0.87	2.90 ± 0.88	0.160
	Softness	3.13 ± 0.67	3.19 ± 0.79	0.618
	Hair loss	3.16 ± 0.90	2.54 ± 0.90	0.005^*^
Scalp skin color	Redness	3.58 ± 0.92	3.79 ± 0.97	0.294

^*^Indicates a significant difference (*p* < 0.05).

According to the data presented in Table 5, no significant differences were observed between men and women in subjective barrier-related assessments, such as perceptions of moisture, glossiness, greasiness, and tightness (*p* > 0.05), which is consistent with the objective instrumental measurements. Regarding microecological evaluation, self-reported hair loss concerns were significantly greater among female participants than male participants. Similarly, female participants rated their dandruff severity higher than male participants, aligning with the objective finding that dandruff area coverage was significantly greater in female participants (*p* < 0.05). In terms of scalp skin color, subjective questionnaire responses indicated no significant gender difference in self-perceived scalp redness, although male participants reported slightly higher values. Objectively, the a^*^ value—reflecting redness—was significantly higher in male participants (*p* < 0.05), showing a consistent trend between subjective and objective measures.

### 3.3 Visual attention study on the characteristics of scalp aging

#### 3.3.1 Results of the eye-tracking instrument

**Figure 4:**
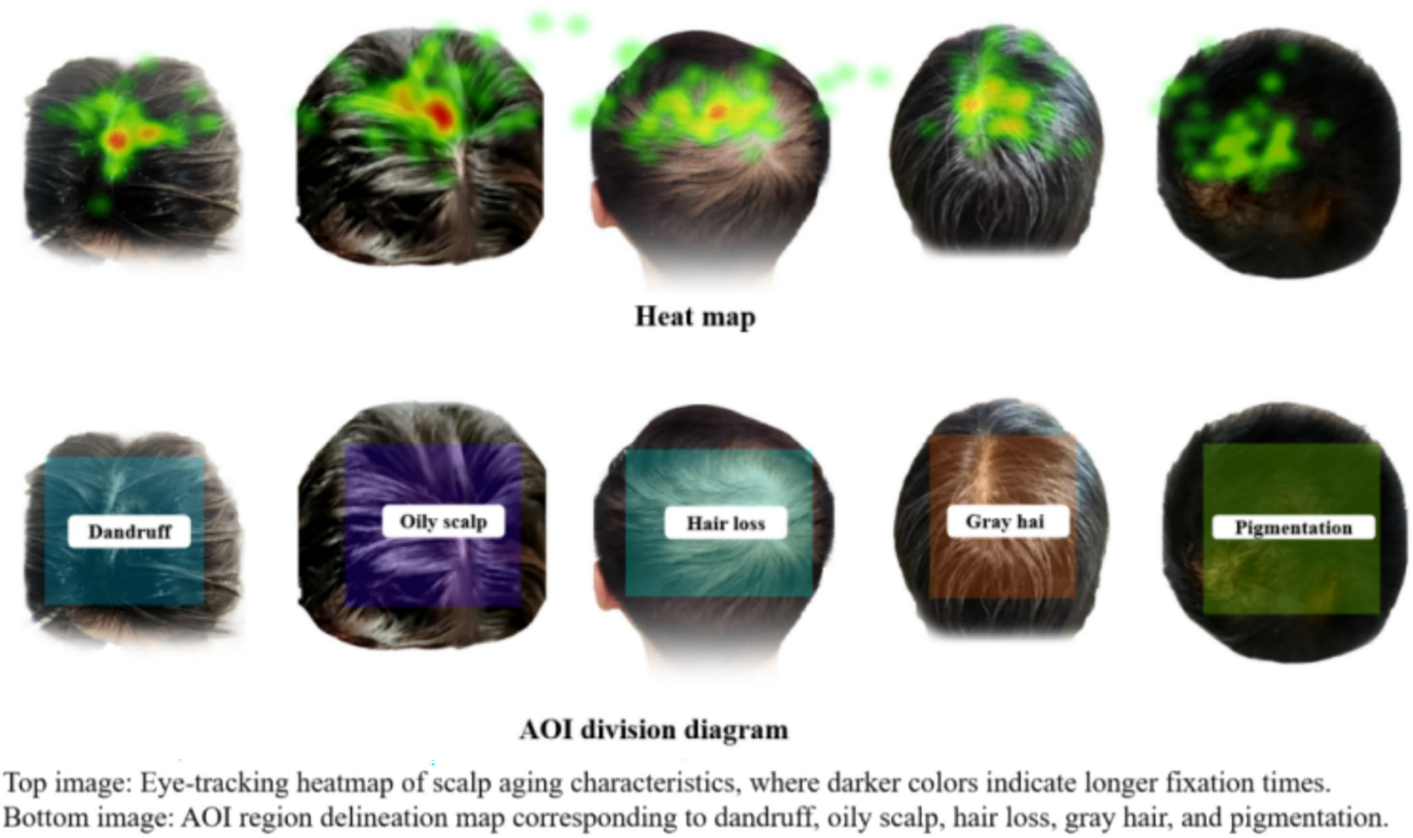
Diagram of the AOI division and an example of a heatmap.

In the AOI analysis conducted using Tobii Pro Lab software, a stimulus image containing five scalp aging features—dandruff, pigmentation, gray hair, oily scalp, and hair loss—was divided into five independent areas of interest (AOIs), with each feature corresponding to one AOI. A schematic diagram of the AOI division and an example of a heatmap are presented in Figure 4. Descriptive results of the related parameters are summarized in Table 6.

**Table 6:** Descriptive statistics of region of interest gaze on scalp aging characteristics.

Gender	Parameters	Characteristics				
		Dandruff	Pigmentation	Gray hair	Oily scalp	Hair loss
Men	Time to first fixation in AOI/s	2.37	4.29	2.79	1.41	2.47
Women		2.68	5.67	4.21^*#^	1.74	1.85
Men	Duration of first fixation in AOI/s	0.45	0.34	0.34	0.29	0.29
Women		0.41	0.37	0.38	0.32	0.26
Men	Total duration of fixation in AOI/s	3.08	2.46	3.48^*#^	3.87	2.65
Women		3.70	2.93	2.87	3.56	3.07
Men	Average duration of fixation in AOI/s	0.43	0.39	0.37	0.35	0.29
Women		0.57^*#^	0.45	0.43	0.46	0.41^*#^
Men	Total duration of visit/s	3.18	2.54	4.04^*#^	4.09	2.73
Women		3.98^*#^	3.03	2.99	3.73	3.25
Men	Number of fixations in AOI/times	8.52	6.90	10.94^*#^	12.19	9.35
Women		9.06	7.68	8.55	11.84	10.26
Men	Number of visits/times	3.81	3.10	5.52	6.35^*#^	5.84
Women		4.17	3.60	4.63	5.06	5.85

^*^Indicates a significant difference (*p* < 0.05).

^#^Indicate a significant difference (ANCOVA, *p* < 0.05).

According to the results in Table 6, significant gender-based differences were observed in visual attention to scalp aging features:

1. Dandruff: Women exhibited significantly longer average fixation duration (0.57 s) and visit duration (3.98 s) compared to men (0.43 s and 3.18 s, respectively; *p* < 0.05), indicating that women allocated more sustained visual attention and cognitive processing to dandruff-related features.
2. Pigmentation: None of the seven parameters showed significant gender differences (*p* > 0.05), suggesting similar levels and patterns of visual attention to pigmentation in both genders, which may be attributed to the relatively low visual salience of this feature on the scalp.
3. Gray hair: Men had a significantly shorter time to first fixation (2.79 s) than women (4.21 s), while demonstrating significantly longer total fixation duration within the AOI (3.48 s), longer visit duration (4.04 s), and higher fixation count within the AOI (10.94) compared to women (2.87 s, 2.99 s, and 8.55, respectively; *p* < 0.05). These results indicate that men responded more quickly to gray hair and engaged in more intensive and frequent visual processing of this feature.
4. Oily scalp: A significant gender difference was observed only in the number of visits to the AOI, with men (6.35) showing significantly higher values than women (5.06; *p* < 0.05), suggesting that men attended more frequently to oily scalp features and incorporated them more often into their visual processing.
5. Hair loss: Women showed a significantly longer average fixation duration (0.41 s) than men (0.29 s; *p* < 0.05), reflecting more sustained visual engagement with hair loss features.

#### 3.3.2 Visual perception questionnaire

To further investigate the visual attention characteristics reflected in the eye-tracking data, this study employed a combination of questionnaires and on-site interviews to supplement the analysis of participants’ visual perception. Significant differences were observed between men and women (*p* < 0.05), with detailed results presented in Table 7.

**Table 7:** Subjective evaluations of scalp aging characteristics by men and women.

Dimensions	Characteristics	Proportion/%	Women		Men		*χ* ^2^	*P*
			Frequency	Proportion/%	Frequency	Proportion/%		
Pay attention to the features first	Dandruff	34.18	16	33.33	11	35.48	0.830	0.934
	Oily scalp	8.86	4	8.33	3	9.68		
	Hair loss	53.16	26	54.17	16	51.61		
	Gray hair	2.53	1	2.08	1	3.23		
	Pigmentation	1.27	1	2.08	0	0.00		
Attractiveness evaluation	Positive	8.86	4	8.33	3	9.68	0.091	0.956
	Negative	69.62	34	70.83	21	67.74		
	Neutral	21.52	10	20.83	7	22.58		
Emotional response	Greatly worried	27.85	13	27.08	9	29.03	0.677	0.713
	A little worried	72.15	34	70.83	23	74.19		
	Do not care	1.27	1	2.08	0	0.00		
First impression influence	Yes	88.61	43	89.58	27	87.10	0.115	0.734
	No	11.39	5	10.42	4	12.90		

The questionnaire results indicated the following:

- First noticed feature: In terms of subjective perception, hair loss was the most frequently first-noticed scalp aging feature among both male and female participants, followed by dandruff. No significant gender difference was observed in the initial feature of attention. This differs from the eye-tracking analysis data, suggesting an inconsistency between initial visual attention and subjective awareness during the search process.
- Attractiveness evaluation: The majority of participants believed that scalp aging features negatively affect attractiveness, with a slightly higher proportion of women holding this view, although the difference was not statistically significant. A small number of participants perceived a bidirectional relationship between scalp aging and attractiveness, while very few considered these features to have a positive impact. These results indicate that scalp aging is widely regarded as a negative aesthetic attribute, potentially influencing others’ evaluations of one’s attractiveness (38).
- Emotional response: The majority of participants reported concern regarding scalp aging, with only a very small minority expressing indifference. No significant gender differences were observed in emotional responses. These results suggest that scalp aging evokes considerable sensitivity and psychological attention, which may be related to societal emphasis on youthful appearance and personal investment in self-image (39).
- Impact on first impression: The majority of participants indicated that scalp aging features could affect first impressions. This finding suggests that scalp appearance may serve as a visual cue in social interactions, thereby influencing judgments about an individual’s physical condition and overall image.

## 4 Conclusion

This study investigated scalp aging characteristics and visual attention patterns in a sample of 79 Han Chinese participants (31 men, 48 women) aged 31–47 years from Shanghai, China, using an integrated approach that combined non-invasive instrumental measurements (scalp physiology), eye-tracking (visual attention), and subjective evaluations (questionnaires). Non-invasive instruments were employed to assess three physiological dimensions: barrier function, microecology, and scalp skin color. Eye-tracking quantified participants’ visual attention to scalp aging features, while subjective evaluations reflected self-perceived scalp conditions and emotional responses. This multi-method approach provides valuable insights into scalp aging differences within the Chinese population.

In terms of scalp physiology: (1) No significant gender differences were observed in scalp barrier function, such as stratum corneum moisture content and TEWL. Subjective assessments were consistent with instrumental measurements. This finding aligns with a study by Darlenski et al. (40), which reported no significant gender-based differences in epidermal barrier function in areas such as the forearm and palm, and our study further confirms that this finding also holds true for the scalp. (2) Significant gender differences were observed in scalp microecology. Women exhibited significantly higher dandruff area coverage and pH than men. Subjectively, women also reported more severe hair loss concerns. Furthermore, self-rated dandruff severity was consistent with instrumental measurements, both indicating more pronounced dandruff issues in women. (3) Regarding scalp skin color, men exhibited significantly higher a^*^ values than women, a result consistent with studies conducted in North Indian (41) and Mexican populations (42), which also reported a tendency toward higher a^*^ values in men. No significant gender difference was observed in b^*^ values. The consistency of these findings across populations reinforces the validity of our conclusions.

In visual attention: Significant gender-based differences were observed in participants’ visual attention to scalp aging features. Women allocated more sustained attention and cognitive resources to dandruff and hair loss, whereas men exhibited more focused and frequent attention to gray hair and oily scalp. These differences may be influenced by sociocultural contexts and individual cognitive habits (43, 44), reflecting distinct visual processing strategies between genders. Subjective questionnaires revealed that 53.16% of participants considered hair loss the most noticeable feature of scalp aging, while 34.18% identified dandruff as the primary concern. However, eye-tracking data indicated that gray hair received the shortest time to first fixation, suggesting that it captures visual attention most rapidly during visual search. This discrepancy between subjective perception and initial gaze behavior may stem from interactions between visual processing strategies and sociocognitive biases, wherein self-construct characteristics also shape an individual’s perception and information processing (45, 46). Furthermore, the majority of participants (69.62%) believed that scalp aging negatively affects attractiveness and expressed high levels of concern (95.95% reported being “Greatly worried” or “A little worried”), highlighting the social significance of scalp appearance in interpersonal interactions.

In conclusion, this study comprehensively evaluated scalp aging characteristics and visual attention patterns in Chinese adults aged 31–47 years using a multi-dimensional methodology. It revealed significant gender-based differences in physiological traits and visual behavior, providing new perspectives on scalp aging and underscoring the value of an integrated assessment approach. However, a limitation of this study is that the sample was restricted to Han Chinese individuals in Shanghai, which may not fully represent other regions or ethnic groups. Future research should expand the sample to include more diverse populations and regions to enhance the generalizability of the findings.

## Data Availability

The original contributions presented in the study are included in the article/supplementary material; further inquiries can be directed to the corresponding author.

## References

[1] Shin SH Lee YH Rho N-K Park KY. Skin aging from mechanisms to interventions: focusing on dermal aging. Front Physiol. (2023) 14:1195272. doi: 10.3389/fphys.2023.1195272, 37234413 PMC10206231

[2] Farage MA Miller KW Elsner P Maibach HI. Characteristics of the aging skin. Adv Wound Care. (2013) 2:5–10. doi: 10.1089/wound.2011.0356, 24527317 PMC3840548

[3] Ganceviciene R Liakou AI Theodoridis A Makrantonaki E Zouboulis CC. Skin Anti-Aging Strategies. Dermatoendocrinol. (2012) 4:308–19. doi: 10.4161/derm.22804, 23467476 PMC3583892

[4] Makrantonaki E Zouboulis CC. The skin as a Mirror of the aging process in the human organism – state of the art and results of the aging research in the German National Genome Research Network 2 (NGFN-2). Exp Gerontol. (2007) 42:879–86. doi: 10.1016/j.exger.2007.07.002, 17689905

[5] Quan T Li R Gao T. Role of mitochondrial dynamics in skin homeostasis: an update. Int J Mol Sci. (2025) 26:1803. doi: 10.3390/ijms26051803, 40076431 PMC11898645

[6] Wong QYA Chew FT. Defining skin aging and its risk factors: a systematic review and meta-analysis. Sci Rep. (2021) 11:22075. doi: 10.1038/s41598-021-01573-z, 34764376 PMC8586245

[7] Chaudhary M Khan A Gupta M. Skin ageing: pathophysiology and current market treatment approaches. Curr Aging Sci. (2020) 13:22–30. doi: 10.2174/1567205016666190809161115, 31530270 PMC7403684

[8] Trueb RM. Oxidative stress in ageing of hair. Int J Trichology. (2009) 1:6–14. doi: 10.4103/0974-7753.51923, 20805969 PMC2929555

[9] Trueb RM Rezende HD Dias MFRG. A comment on the science of hair aging. Int J Trichology. (2018) 10:245–54. doi: 10.4103/ijt.ijt_56_18, 30783331 PMC6369639

[10] Tobin DJ. Aging of the hair follicle pigmentation system. Int J Trichol. (2009) 1:83–93. doi: 10.4103/0974-7753.58550, 20927229 PMC2938584

[11] Boggio PS Wingenbach TSH Da Silveira Coêlho ML Comfort WE Marques LM Alves MVC. Social and affective neuroscience of everyday human interaction: from theory to methodology. Berlin: Springer International Publishing (2023). 37988484

[12] Hessels RS Hooge ITC. Eye tracking in developmental cognitive neuroscience – the good, the bad and the ugly. Dev Cogn Neurosci. (2019) 40:100710. doi: 10.1016/j.dcn.2019.100710, 31593909 PMC6974897

[13] Zhu L Chen J Yang H Zhou X Gao Q Loureiro R . Wearable near-eye tracking technologies for health: a review. Bioengineering. (2024) 11:738. doi: 10.3390/bioengineering11070738, 39061820 PMC11273595

[14] Lim JZ Mountstephens J Teo J. Emotion recognition using eye-tracking: taxonomy, review and current challenges. Sensors. (2020) 20:2384. doi: 10.3390/s20082384, 32331327 PMC7219342

[15] Liu H Liu H Li F Han B Wang C. Effect of cognitive control on attentional processing of emotional information among older adults: evidence from an eye-tracking study. Front Aging Neurosci. (2021) 13:644379. doi: 10.3389/fnagi.2021.644379, 33994995 PMC8116557

[16] Guo R Kim N Lee J. Empirical insights into eye-tracking for design evaluation: applications in visual communication and new media design. Behav Sci (Basel). (2024) 14:1231. doi: 10.3390/bs14121231, 39767372 PMC11673074

[17] Cvahte Ojsteršek T Topolšek D. Eye tracking use in researching driver distraction: a scientometric and qualitative literature review approach. J Eye Mov Res. (2019) 12:1–30. doi: 10.16910/jemr.12.3.5 PMC7880134 33828732

[18] Zammarchi G Conversano C. Application of eye tracking technology in medicine: a bibliometric analysis. Vision (Basel). (2021) 5:56. doi: 10.3390/vision5040056, 34842855 PMC8628933

[19] Srivastava P Ramakanth D Akhila K Gaikwad KK. Package design as a branding tool in the cosmetic industry: consumers’ perception vs. reality. SN Bus Econ. (2022) 2:58. doi: 10.1007/s43546-022-00222-5, 35615336 PMC9123395

[20] Maghsoudi Rahim Abadi M Keimasi M Abedi E. The influence of consumers’ visual attention on product packaging elements in their purchasing process using eye-tracking technology. BMJ Open. (2025) 2:73–88. doi: 10.61838/bmfopen.2.1.8

[21] Ko E-S Kim JN Na HJ Kim ST. Changes in pupil size according to the color of cosmetic packaging: using eye-tracking techniques. Appl Sci. (2024) 15:73. doi: 10.3390/app15010073

[22] Wu W Lin J Yang R. Chinese expert consensus on scalp anti-aging. J Pract Dermatol. (2020) 13:321–5. doi: 10.11786/sypfbxzz.1674-1293.20200601

[23] Lee YI Kim J Park SR Ham S Lee HJ Park CR . Age-related changes in scalp biophysical parameters: a comparative analysis of the 20s and 50s age groups. Skin Res Technol. (2023) 29:e13433. doi: 10.1111/srt.13433, 37632187 PMC10408001

[24] Williams R Westgate GE Pawlus AD Sikkink SK Thornton MJ. Age-related changes in female scalp dermal sheath and dermal fibroblasts: how the hair follicle environment impacts hair aging. J Invest Dermatol. (2021) 141:1041–51. doi: 10.1016/j.jid.2020.11.009, 33326808

[25] Maymone MBC Laughter M Pollock S Khan I Marques T Abdat R . Hair aging in different races and ethnicities. J Clin Aesthetic Dermatol. (2021) 14:38–44. doi: 10.2340/1901-7349-3213 PMC7869811 33584967

[26] Schild J Kalvodová A Zbytovská J Farwick M Pyko C. The role of ceramides in skin barrier function and the importance of their correct formulation for skincare applications. Int J Cosmet Sci. (2024) 46:526–43. doi: 10.1111/ics.12972, 39113291

[27] Alexander H Brown S Danby S Flohr C. Research techniques made simple: Transepidermal water loss measurement as a research tool. J Invest Dermatol. (2018) 138:2295–2300.e1. doi: 10.1016/j.jid.2018.09.001, 30348333

[28] Watanabe K Nishi E Tashiro Y Sakai K. Mode and structure of the bacterial community on human scalp hair. Microbes Environ. (2019) 34:252–9. doi: 10.1264/jsme2.ME19018, 31217363 PMC6759350

[29] Watanabe K Yamada A Nishi Y Tashiro Y Sakai K. Relationship between the bacterial community structures on human hair and scalp. Biosci Biotechnol Biochem. (2020) 84:2585–96. doi: 10.1080/09168451.2020.1809989, 32993459

[30] Rasheedkhan Regina V Chopra T Weihao K Cheruvalli S Sabrina A Jamal Mohamed HF . Decoding scalp health and microbiome dysbiosis in dandruff. Microbiology. (2024). doi: 10.1101/2024.05.02.592279

[31] Boer M Duchnik E Maleszka R Marchlewicz M. Structural and biophysical characteristics of human skin in maintaining proper epidermal barrier function. Adv Dermatol Allergol. (2016) 33:1–5. doi: 10.5114/pdia.2015.48037, 26985171 PMC4793052

[32] Mayser P Genrich F Meunier L Nordzieke S. Scalp microbiome and dandruff—exploring novel biobased esters. Cosmetics. (2024) 11:174. doi: 10.3390/cosmetics11050174

[33] Man MQ Xin SJ Song SP Cho SY Zhang XJ Tu CX . Variation of skin surface pH, sebum content and stratum corneum hydration with age and gender in a large Chinese population. Skin Pharmacol Physiol. (2009) 22:190–9. doi: 10.1159/000231524, 19648780 PMC2836947

[34] Kim MK Choi SY Byun HJ Patel RA Shinn AH Huh CH . Evaluation of gender difference in skin type and pH. J Dermatol Sci. (2006) 41:153–6. doi: 10.1016/j.jdermsci.2005.12.001, 16406502

[35] Itou T Ito S Wakamatsu K. Effects of aging on hair color, melanosomes, and melanin composition in Japanese males and their sex differences. Int J Mol Sci. (2022) 23:14459. doi: 10.3390/ijms232214459, 36430936 PMC9693441

[36] Del Bino S Duval C Bernerd F. Clinical and biological characterization of skin pigmentation diversity and its consequences on UV impact. Int J Mol Sci. (2018) 19:2668. doi: 10.3390/ijms19092668, 30205563 PMC6163216

[37] Taylor S Westerhof W Im S Lim J. Noninvasive techniques for the evaluation of skin color. J Am Acad Dermatol. (2006) 54:S282–90. doi: 10.1016/j.jaad.2005.12.041 16631969

[38] Åberg E Kukkonen I Sarpila O. From double to triple standards of ageing. Perceptions of physical appearance at the intersections of age, gender and class. J Aging Stud. (2020) 55:100876. doi: 10.1016/j.jaging.2020.100876, 33272447

[39] Dhami L. Psychology of hair loss patients and importance of Counseling. Indian J Plast Surg. (2021) 54:411–5. doi: 10.1055/s-0041-1741037, 34984078 PMC8719979

[40] Darlenski R Fluhr JW. Influence of skin type, race, sex, and anatomic location on epidermal barrier function. Clin Dermatol. (2012) 30:269–73. doi: 10.1016/j.clindermatol.2011.08.013, 22507039

[41] Dabas P Nayak BP Khajuria H Jain S Dutt S Saraswathy KN. A cross-sectional assessment of quantitative epidermal melanin and erythema indices among north Indians. Indian Dermatol Online J. (2023) 14:366–70. doi: 10.4103/idoj.idoj_400_22, 37266078 PMC10231713

[42] Castanedo-Cazares JP Hernández-Blanco D García-Cortés JD Medina-Aguilar L Torres-Álvarez B. Análisis de la pigmentación cutánea en una muestra. Gac Med Mex. (2018) 154:449. doi: 10.24875/GMM.17003050 29420524

[43] Čeněk J Halámková D Caha J Lacko D Kalenská P Stachoň Z . Cross-cultural analysis of eye-movement patterns in visual scene perception: a comparison of seven cultural samples. Sci Rep. (2025) 15:28574. doi: 10.1038/s41598-025-12724-x, 40764356 PMC12326012

[44] Hwang YM Lee KC. Using an eye-tracking approach to explore gender differences in visual attention and shopping attitudes in an online shopping environment. Int J Human-Comput Interact. (2018) 34:15–24. doi: 10.1080/10447318.2017.1314611

[45] Bicknell K Levy R Rayner K. Ongoing cognitive processing influences precise eye-movement targets in Reading. Psychol Sci. (2020) 31:351–62. doi: 10.1177/0956797620901766, 32105193 PMC7436780

[46] Taragin D Tzuriel D Vakil E. Mental rotation: the effects of processing strategy, gender and task characteristics on children’s accuracy, reaction time and eye movements’ pattern. J Eye Mov Res. (2019) 12:2–19. doi: 10.16910/jemr.12.8.2 PMC7881899 33828779

